# Borderline personality disorder and decision-making capacity

**DOI:** 10.1192/j.eurpsy.2022.1713

**Published:** 2022-09-01

**Authors:** F. Garcia Lazaro

**Affiliations:** Hospital Virgen del Rocío Sevilla, Ugc Salud Mental, Sevilla, Spain

**Keywords:** borderline, legal, personality, disorder

## Abstract

**Introduction:**

Borderline personality disorder is characterized by a pattern in which instability in interpersonal relationships, self-image and affections prevails, and intense impulsivity present in the early stages of adulthood and with altered functionality in several contexts.

**Objectives:**

Establish what functions may be altered in crisis situations in borderline personality disorder.

Point out what legal tools we have available in situations in which the will is altered in borderline personality disorder.

Reflect on borderline personality disorder and its relationship with substance use.

**Methods:**

Regarding a clinical case with a 25-year-old patient with a diagnosis of Borderline Personality Disorder and a history of use in a pattern of dependence (opioids, cannabis, cocaine) who is admitted to a hospital for diagnostic and therapeutic procedures secondary to pathology to which it is denied, determining the absence of the capacity to give consent. A systematic review of the existing bibliography on borderline personality disorder, substance use disorder and decision-making capacity has been carried out using as key words: borderline personality disorder decision-making capacity.

**Results:**

The presence of anxious symptoms, affective instability, feelings of emptiness and hopelessness as well as impulsivity can give rise to scenarios in which the decision-making capacity is impaired, being necessary to resort to legal means that allow us to prioritize the well-being of the patient.

**Conclusions:**

The decision-making capacity can be altered in crisis situations in borderline personality disorder, having legal tools at hand that allow us to carry out actions to preserve the physical state of patients.

**Disclosure:**

No significant relationships.

